# *Zeb1* Regulates the Function of Lympho-Myeloid Primed Progenitors after Transplantation

**DOI:** 10.3390/biom13091386

**Published:** 2023-09-14

**Authors:** Alhomidi Almotiri, Ashleigh S. Boyd, Neil P. Rodrigues

**Affiliations:** 1 Department of Clinical Laboratory Sciences, College of Applied Medical Sciences-Dawadmi, Shaqra University, Dawadmi 17464, Saudi Arabia; hsalmutiri@su.edu.sa; 2 European Cancer Stem Cell Research Institute, School of Biosciences, Cardiff University, Hadyn Ellis Building, Cardiff CF24 4HQ, UK; 3 Department of Surgical Biotechnology, Division of Surgery and Interventional Science, Royal Free Hospital, University College London, London NW3 2PS, UK; a.boyd@ucl.ac.uk; 4 Institute of Immunity and Transplantation, University College London, London NW3 2PP, UK

**Keywords:** *Zeb1*, hematopoiesis, differentiation

## Abstract

*Zeb1*, a zinc finger E-box binding homeobox epithelial–mesenchymal (EMT) transcription factor, acts as a critical regulator of hematopoietic stem cell (HSC) self-renewal and multi-lineage differentiation. Whether *Zeb1* directly regulates the function of multi-potent progenitors primed for hematopoietic lineage commitment remains ill defined. By using an inducible *Mx-1 Cre* conditional mouse model where *Zeb1* was genetically engineered to be deficient in the adult hematopoietic system (hereafter *Zeb1^−/−^*), we found that the absolute cell number of immunophenotypically defined lympho-myeloid primed progenitors (LMPPs) from *Zeb1^−/−^* mice was reduced. Myeloid- and lymphoid-biased HSCs in *Zeb1^−/−^* mice were unchanged, implying that defective LMPP generation from *Zeb1^−/−^* mice was not directly caused by an imbalance of lineage-biased HSCs. Functional analysis of LMPP from *Zeb1^−/−^* mice, as judged by competitive transplantation, revealed an overall reduction in engraftment to hematopoietic organs over 4 weeks, which correlated with minimal T-cell engraftment, reduced B-cell and monocyte/macrophage engraftment, and unperturbed granulocyte engraftment. Thus, *Zeb1* regulates LMPP differentiation potential to select lympho-myeloid lineages in the context of transplantation.

## 1. Introduction

The daily production of blood cells—termed hematopoiesis—involves a rare master cell type, hematopoietic stem cells (HSCs), that generate intermediate progenitors, which divide further and eventually become restricted to specific myeloid and lymphoid blood cells responsible for the provision of essential physiologic processes, including the resolution of infection, inflammation, tumor immunosurveillance, oxygen transport, and clotting [1]. For hematopoiesis to occur without flaw, HSCs are highly regulated in a cell-intrinsic manner by transcription factors (TFs) and extrinsically by the bone marrow microenvironment that they inhabit [2]. Within this complicated regulatory framework, the genetic and epigenetic integrity of HSCs is protected by cell cycle/apoptotic checkpoints [3] and is balanced by the overall need for rare HSCs to self-renew in order to sustain their activity during life and to differentiate to produce blood in times of physiologic need [4,5].

Epithelial–mesenchymal transition (EMT) is involved in several cellular contexts in embryonic development, regeneration, and adult tissue maintenance, where epithelial cells relinquish their cell polarity and cell adhesion characteristics while increasing their migratory capacity and acquiring mesenchymal cell properties [6]. EMT is regulated by specific EMT TFs that include the ZEB, SNAI, and TWIST families of TFs [7]. *Zeb1*, a zinc finger E-box binding homeobox TF, regulates EMT in gastrulation, myogenesis, and neurogenesis in normal tissue development and maintenance [8]. In the setting of cancer, the aberrant regulation of EMT occurs, increasing stem cell properties and migration alike, with both processes encouraging tumor progression through metastasis, and with ‘stemness’ leading to therapy resistance [9]. In this respect, there is ample evidence to demonstrate that *Zeb1* confers characteristics of ‘stemness’, including self-renewal, and increases invasiveness in cancer [8,10].

It is becoming increasingly appreciated that EMT TFs are paramount to tissue maintenance beyond epithelial tissues, with *Zeb1* being an exemplar of such regulation in the hematopoietic system [7,11]. For example, *Zeb1* is required for cell-intrinsic T-cell development [12]. Additionally, we and others have shown that *Zeb1* acts as a pivotal regulator of HSC self-renewal and, besides T-cell differentiation, *Zeb1* functions as an essential regulator of multi-lineage differentiation in hematopoiesis [11,13,14].

Using a genetically engineered mouse that contains ‘floxed’ alleles of *Zeb1* and an inducible *Mx-1-Cre* [15], wherein *Zeb1* expression can be removed specifically in adult hematopoietic cells by administering polyinosinic–polycytidylic acid (pIpC), we previously established that defective myeloid and lymphoid differentiation maps in *Zeb1*^−/−^ mice to a defect in HSCs and multi-potent progenitors primed for commitment to lympho-myeloid lineages, so-called lympho-myeloid-primed progenitors (LMPPs) [11]. However, the extent to which *Zeb1* is specifically required for functional LMPP differentiation in vivo remains unclear and is addressed in our current study by the functional analysis of Zeb1-deficient LMPPs in competitive transplantation experiments, where we find that Zeb1 mediates LMPP differentiation to T-cell, B-cell, and monocyte/macrophage lineages but is expendable for the regulation of granulocyte differentiation in vivo.

## 2. Materials and Methods

### 2.1. Mice

We utilized *Zeb1^fl^*^/fl^ mice [15], which were bred with *Mx1-Cre*^+/−^ mice, to generate an experimental cohort of *Zeb1^f^*^l/fl^;*Mx1-Cre*^−/−^ (control) and *Zeb1*^fl/fl^;*Mx1-Cre*^+/−^ (*Zeb1^−/−^*). *Zeb1* was deleted after the intraperitoneal (IP) administration of polyinosinic–polycytidylic acid (pIpC) (6 doses every alternate day, 0.3 mg per dose, GE Healthcare). All experiments were performed under the legal authority of the UK Home Office.

### 2.2. Flow Cytometry Analysis

Flow cytometry analysis of hematopoietic cells was performed according to previously published protocols [16]. Bones (femurs, tibias, iliac bones) were crushed using a pestle and mortar in phosphate-buffered saline (PBS) supplemented with 2% fetal bovine serum (FBS) and the BM cell suspension was filtered through a 70 μm cell strainer (Miltenyi Biotec, Bergisch Gladbach, Germany). Spleen and thymi were homogenized through a 70 μm cell strainer. PB was obtained by tail vein bleeding and blood was collected in EDTA-treated tubes (Starstedt, Nümbrecht, Germany). Red blood cells were lysed from PB, spleen, and BM by ammonium chloride solution (StemCell Technologies, Vancouver, BC, Canada). For the immunophenotypic analysis of HSC and LMPPs, the following antibodies were utilized for staining: a lineage cocktail mix was prepared from a selection of biotin antibodies for lineage cell markers in PBS 2% FBS (MAC1 and GR1 for myeloid cells, TER119 for erythroid lineage, B220 for B cells, and CD3e, CD4, CD8a for T cells), and cells were stained with these in addition to SCA-1, C-KIT, CD150, CD48, CD135, and CD34, where HSC is defined as lineage-negative SCA-1^+^ C-KIT^+^ CD150^+^ CD48^−^, and LMPP is defined as lineage-negative SCA-1^+^ C-KIT^+^ CD135^high^ CD34^+^. Biotin lineage antibodies were detected by the addition of a streptavidin-conjugated fluorochrome. For lineage-positive cell analysis of the BM and spleen, cells were stained for GR1 and MAC1 (myeloid cells: monocytes and granulocytes), CD3, CD4 and CD8 (T cells), and B220 (B cells). Thymocytes were stained for CD4 and CD8, CD44, CD25 and C-KIT to study early and late stages of T-cell development in the thymus. Anti-CD45.1 and anti-CD45.2 were used in the staining mix to differentiate between donor and recipient cells. Samples were analyzed using BD LSRFortessa^TM^ (BD Biosciences, Becton, NJ, USA). Data were analyzed using FlowJo 10.0.8 (Tree Star, Inc., Ashland, OR, USA).

### 2.3. LMPP Fluorescence-Activated Cell Sorting (FACS)

Fluorescence-activated cell sorting (FACS) was carried out according to a previously published protocol [17]. For LMPP (Lineage^−^ SCA-1^+^ C-KIT^+^ CD135^high^ CD34^+^), a BM cell suspension was obtained, and red blood cells were lysed by ammonium chloride solution (StemCell Technologies). Cells were enriched for C-KIT by magnetic-activated cell sorting (MACS) (MACS^®^, Miltenyi Biotec, Bergisch Gladbach, Germany) using anti-CKIT magnetic beads (Miltenyi Biotec). CKIT^+^ cells were stained as follows: a lineage cocktail was prepared from a pool of biotin antibodies recognizing lineage-specific cell markers in PBS 2% FBS (MAC1 and GR1 for myeloid cells, TER119 for erythroid lineage, B220 for B cells, CD3e, CD4, CD8a for T cells), SCA1-APCCy7, CKIT-APC, CD34-FITC, and CD135-PE. The lineage cocktail was detected by the addition of a streptavidin-conjugated fluorochrome. LMPPs were sorted using a BD FACSAria^TM^ Fusion (BD Biosciences).

### 2.4. Transplantation Experiments

Transplantation experiments were used to assess the functional potential of LMPPs in vivo. C57BL/6 SJL mice (CD45.1) were used as recipients. Mice were lethally irradiated at 9 Gy (split dose). For LMPP transplantation, 2000 LMPPs from *Zeb1^−/−^* and control mice (CD45.2) mixed with 1.4 × 10^5^ whole BM (CD45.1) (supporting cells) were intravenously transplanted into lethally irradiated mice (CD45.1). To monitor the engraftment, tail vein bleeding was performed at weeks 1, 2, 3, 4 post-transplantation and flow cytometry analysis for engraftment for CD45.1 (recipient/competitor) and CD45.2 (donor LMPP) was performed together with lineage-positive cell analysis as described in Section 2.2. Full experimental details of competitive transplantation have been described previously [18].

### 2.5. Statistical Analysis

Figures were prepared using Prism (GraphPad Software, Inc., Boston, MA, USA). Statistical analyses were performed using the Mann–Whitney U test to calculate significance as follows: * *p* < 0.05, ** *p* < 0.01, *** *p* < 0.001, **** *p* < 0.0001.

## 3. Results

### 3.1. Acute Conditional Deletion of Zeb1 in Hematopoietic Cells Causes a Near Reduction in the Absolute Number of LMPPs Independently of Lineage-Biased HSCs

**Figure 1:**
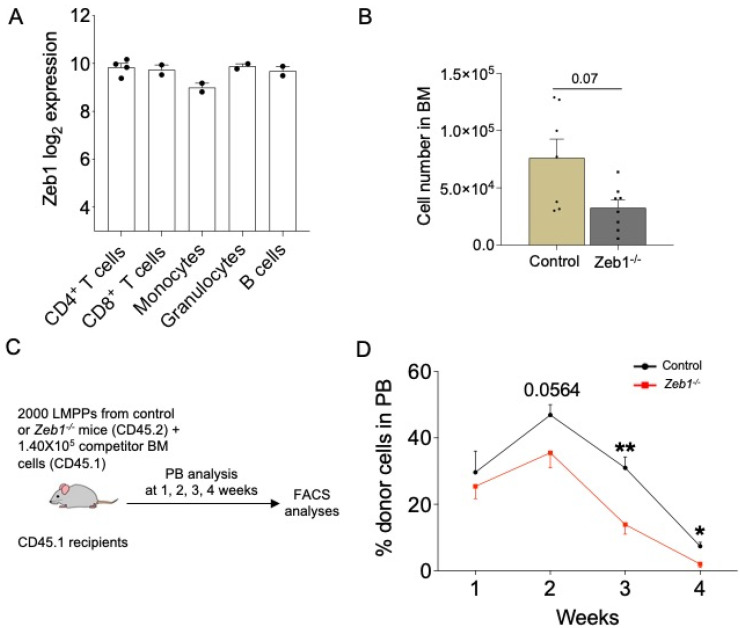
**Acute conditional loss of *Zeb1* results in a peripheral blood engraftment defect after LMPP transplantation.** (**A**) *Zeb1* log2 expression data in subsets of mature blood cells. Data from BloodSpot. (**B**) Cell number of LMPPs (LSK CD34^+^ CD135^high^) in BM from control (*n* = 7) and *Zeb1^−/−^* (*n* = 8) mice 14 days after the last dose of pIpC from 4 independent experiments. (**C**) A scheme of the LMPP transplantation. Two thousand LMPPs from control or *Zeb1^−/−^* mice (donor CD45.2) mixed with 1.40 × 10^5^ BM competitor cells (CD45.1) were transplanted into lethally irradiated recipients (CD45.1) and the mice were monitored by bleeding the tail vein at week 1, 2, 3, and 4. (**D**) The percentage of donor cells in PB at weeks 1, 2, 3, 4 post LMPP transplantation from control (*n* = 9–10, week 4 *n* = 5) and *Zeb1^−/−^* (*n*= 9–10, week 4 *n* = 5) mice from 2 independent experiments, except week 4 from one experiment. Error bars show mean ± SEM. Mann–Whitney U test was used to calculate significance as follows: * *p* < 0.05, ** *p* < 0.01.

*Zeb1* is known to regulate select myeloid lineages and T-lymphoid differentiation in mice [11], yet relative *Zeb1* expression between all major myeloid and lymphoid lineages remains equivalent, as judged by bioinformatic analysis of the BloodSpot database (www.bloodspot.eu, accessed on 6 June 2023) (Figure 1A). We therefore asked whether *Zeb1* regulates lympho-myeloid differentiation at the incipient stage of lineage commitment rather than the later-stage maturation of these lineages. To this end, we sought to evaluate the specific requirement for *Zeb1* in LMPPs, which are the earliest isolatable lympho-myeloid-committed progenitors [19]. Using the principle of conditional mouse genetics, we mated mice engineered to have ‘floxed’ alleles of *Zeb1* (*Zeb1^fl/fl^* mice) [15] to *Mx1-Cre^+^* mice [20] that generated either *Zeb ^fl/fl^;Mx1-Cre^+/−^* or control (*Zeb1 ^fl/fl^;Mx1-Cre^−/−^)* offspring and which were given pIpC every other day for 10 days to achieve the deletion of *Zeb1* in the hematopoietic system (hereafter referred to as *Zeb1^−/−^*). We assessed hematopoiesis in control or *Zeb1^−/−^* mice 14 days after the last dose of pIpC was administered. Full deletion of *Zeb1* was achieved in the LMPP compartment, as previously demonstrated [11]. By immunophenotyping (gating strategy shown in Supplementary Figure S1), we evaluated LMPP abundance in control or *Zeb1^−/−^* mice and found that while LMPPs were not reduced in frequency [11], they were near significantly reduced in absolute number in *Zeb1^−/−^* mice (Figure 1B). Accumulating evidence suggests that while HSCs have the capacity to form all blood lineages, the HSC compartment is genetically, epigenetically, and functionally heterogenous with HSC clones that contribute to hematopoiesis having either myeloid or lymphoid bias or balanced myeloid/lymphoid potential [21]. We therefore asked if the reduction in the absolute number of LMPP in *Zeb1^−/−^* mice was merely due to alterations in lineage-biased HSCs, judged by CD150 expression within the HSC compartment (Lin^−^Sca-1^+^ckit^+^CD48^−^CD150^+^) [22], and found no evidence for changes in the frequency or absolute number of CD150^lo^ (lymphoid-biased HSCs), CD150^med^ (lineage-balanced HSCs), or CD150^hi^ (myeloid-biased HSCs) HSCs in *Zeb1^−/−^* mice (Supplementary Figure S2). Thus, we infer that the reduction in LMPPs from *Zeb1^−/−^* mice was not caused by the disproportionate representation of myeloid- versus lymphoid-biased HSCs in *Zeb1^−/−^* mice.

### 3.2. Acute Conditional Deletion of Zeb1 in Hematopoietic Cells Leads to an Overall Reduction in Engraftment Potential of LMPP-Derived Blood Cells after Transplantation

While LMPPs were reduced in abundance immunophenotypically in *Zeb1^−/−^* mice, it remained unclear how this reflected their differentiation capacity in vivo. To directly test the functionality of LMPPs from *Zeb1^−/−^* mice, we employed competitive transplantation experiments [19] where we prospectively isolated 2000 LMPPs (CD45.2) from control or *Zeb1^–/–^* mice at 14 days following pIpC-induced deletion of *Zeb1*, admixed these cells with untreated 1.4 × 10^5^ BM competitor cells (CD45.1), and intravenously transplanted the mixture of cells into lethally irradiated recipients (CD45.1) (Figure 1C). To gauge LMPP differentiation in vivo after transplantation, the overall contribution of donor CD45.2 LMPP cells to recipient CD45.1 peripheral blood (PB) was distinguished and measured on a weekly basis for 4 weeks using flow cytometry. At week 1 after transplantation, no significant difference was noted in LMPP engraftment potential between the two genotypes (Figure 1D). However, over time we found that there was a significant, gradual erosion of donor cell engraftment to PB in recipients receiving LMPPs from the *Zeb1^−/−^* genotype, indicating that *Zeb1* mediates LMPP differentiation in vivo in the setting of transplantation.

### 3.3. No T-Cell Engraftment from Zeb1^−/−^ LMPPs after Transplantation Due to Impact on T-Cell Maturation

**Figure 2:**
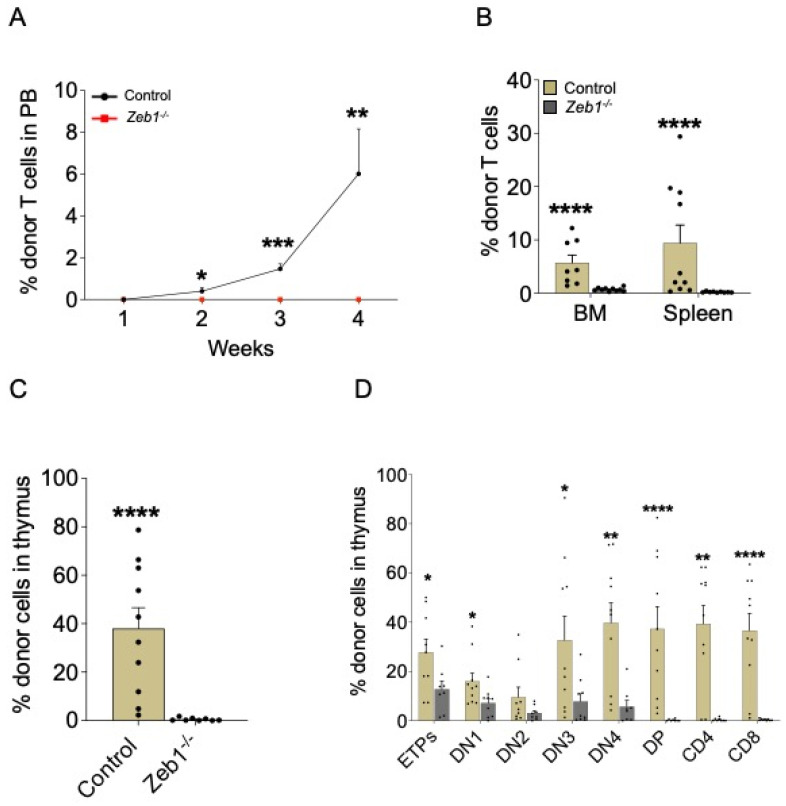
**Acute conditional loss of *Zeb1* impacts T-cell development and maturation after LMPP transplantation.** (**A**) Analysis of PB donor contribution to T cells (CD4^+^/CD8^+^) post LMPP transplantation from control (*n* = 9–10, week 4 *n* = 5) and *Zeb1^−/−^* (*n* = 9–10, week 4 *n* = 5) mice from 2 independent experiments. (**B**) Analysis of BM and spleen donor contribution to T cells (CD4^+^/CD8^+^) 3–4 weeks post LMPP transplantation from control (*n* = 9–10) and *Zeb1^−/−^* (*n* = 9–10) mice from 2 independent experiments. The percentage of donor cells in thymus (**C**) and donor contribution to T-cell populations in thymus (**D**) 3–4 weeks post LMPP transplantation from control (*n* = 9–10) and *Zeb1^−/−^* (*n* = 9–10) mice from 2 independent experiments. Error bars show mean ± SEM. Mann–Whitney U test was used to calculate significance as follows: * *p* < 0.05, ** *p* < 0.01, *** *p* < 0.001, **** *p* < 0.0001.

To appraise which specific blood and immune cell lineages are mediated by *Zeb1* during LMPP differentiation in vivo, we conducted an analysis of specific PB lineages in transplant recipients on a weekly basis by flow cytometry, and, on conclusion of the experiment at week 4, we comprehensively assessed engraftment in hematopoietic organs—namely bone marrow (BM), spleen, or thymus. Given the essential role of *Zeb1* in T-cell generation [12], we commenced our analysis by examining T-cell engraftment in recipients receiving LMPPs from control or *Zeb1^−/−^* donors. T cells derived from *Zeb1^−/−^* LMPPs failed to contribute to recipient hematopoiesis, contrasting strikingly with T-cell engraftment from control LMPPs, which gradually increased over time (Figure 2A). This result was recapitulated in the BM, spleen, and thymus (Figure 2B,C), suggesting that *Zeb1* functionally mediates T-cell development, at least in part, through LMPP differentiation. To further delineate how *Zeb1* regulates T-cell maturation from LMPPs, we assessed T-cell development in the thymus at the incipient stages of T-cell development (in early thymic progenitors: ETPs), through negative selection (in double-negative cell populations: DN1-4), positive selection (in the double-positive population: DP), and, finally, the production of mature CD4^+^ and CD8^+^ T cells. Almost all stages of T-cell development were significantly reduced in recipients engrafted with *Zeb1^−/−^* LMPPs (Figure 2D). Notably, ETPs derived from recipients receiving LMPPs from the *Zeb1^−/−^* genotype were significantly reduced, demonstrating that LMPP-derived progenitors seeding the thymus were defective. During negative selection, the engraftment of DN populations from *Zeb1^−/−^* LMPPs dwindled further, and, in the transition to positive selection, DP engraftment was almost entirely extinguished in the recipient group receiving *Zeb1^−/−^* LMPPs, with the consequence that mature CD4^+^ and CD8^+^ T cells were detected at extremely low levels in these recipients (Figure 2D). Thus, our data demonstrate the functional requirement for *Zeb1* in mediating T-cell development from LMPPs in vivo.

### 3.4. Reduced B-Cell and Monocyte/Macrophage Lineage Potential, but Unimpaired Granulocytic Differentiation from Zeb1^−/−^ LMPPs after Transplantation

**Figure 3:**
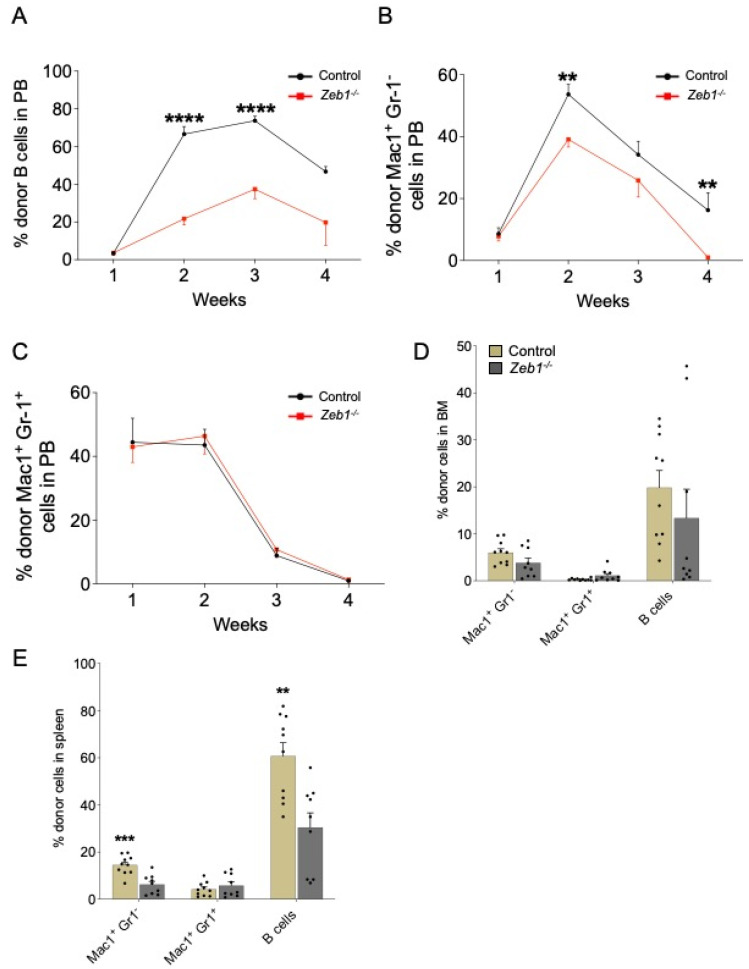
**Acute conditional loss of *Zeb1* results in a B-cell and monocyte/macrophage differentiation defect after LMPP transplantation.** Analysis of PB donor contribution to B cells (B220^+^) (**A**), Mac1^+^ Gr-1^−^ (**B**), Mac1^+^ Gr-1^+^ (**C**) post LMPP transplantation from control (*n* = 9–10, week 4 *n* = 5) and *Zeb1*^−/−^ (*n* = 9–10, week 4 *n* = 5) mice from 2 independent experiments, except week 4 from one experiment. (**D**) Percentage of donor cells in BM and donor contribution to B cells (B220^+^), Mac1^+^ Gr-1^−^, and Mac1^+^ Gr-1^+^ post LMPP transplantation from control (*n* = 9–10) and *Zeb1*^−/−^ (*n* = 9–10) mice from 2 independent experiments. (**E**) Percentage of donor cells in spleen and donor contribution to B cells (B220^+^), Mac1^+^ Gr-1^−^, and Mac1^+^ Gr-1^+^ post LMPP transplantation from control (*n* = 9–10) and *Zeb1*^−/−^ (*n* = 9–10) mice from 2 independent experiments. Error bars show mean ± SEM. Mann–Whitney U test was used to calculate significance as follows: ** *p* < 0.01, *** *p* < 0.001, **** *p* < 0.0001.

Next, we examined B-lymphopoiesis in recipients of LMPPs derived from either control or *Zeb1^−/−^* mice. PB engraftment of LMPPs to the B-cell lineage was reduced in recipients of the *Zeb1^−/−^* genotype over 4 weeks of analysis (Figure 3A). The engraftment of B cells was selectively impaired in the spleen but not BM, pointing to a specific impact of *Zeb1* on extramedullary B-cell maturation from LMPPs (Figure 3D,E). In the myeloid compartment, *Zeb1^−/−^* LMPP-derived Mac-1^+^Gr-1^−^ cells, consistent with the monocyte lineage [23,24], decreased in the PB over time, whereas granulocytic differentiation, marked by Mac-1^−^Gr-1^+^ cells [23,24], was comparable between recipients receiving LMPPs from the two genotypes (Figure 3B,C). *Zeb1^−/−^* LMPP-derived Mac-1^+^Gr-1^−^ macrophage cells contributed less to spleen engraftment than their control counterparts but were unchanged in the BM (Figure 3D,E). In contrast, Mac-1^−^Gr-1^+^ granulocytes derived from LMPP displayed similar BM and splenic engraftment in both control and *Zeb1^−/−^* groups (Figure 3D,E). Thus, *Zeb1* does not appear to be a global regulator of myeloid lineages derived from LMPPs in vivo, but rather regulates the select differentiation of LMPPs to the monocyte/macrophage lineage after transplantation.

## 4. Discussion

*Zeb1* has emerged as a critical regulator of HSC self-renewal and lympho-myeloid lineage differentiation [11], yet little is known about how multi-potent progenitor subsets, the immediate progeny of HSCs, contribute to this *Zeb1*-mediated differentiation defect. In this report, we explored the role of *Zeb1* in a population of multi-potent progenitors at the earliest stage of lympho-myeloid commitment, LMPPs, and found that the acute deletion of *Zeb1* in LMPPs reduced both their absolute number and differentiation capacity to lympho-myeloid lineages in the context of competitive transplantation assays. Thus, we have identified a requirement for *Zeb1* in mediating LMPP differentiation potential in vivo.

In our study, we utilized an inducible conditional mouse model using *Mx1-Cre*, where *Zeb1* expression was deleted in adult HSCs and all their descendants. Thus, in principle, the observed functional impact of *Zeb1^−/−^* LMPPs in transplantation may simply be a read-out of altered transcriptional programming that is hardwired from *Zeb1^−/−^* HSCs [11], with attendant impacts on lineage bias in HSC clones, which are ultimately conveyed to multi-potent progenitors during HSC differentiation. Arguing against this notion, however, we found parity in the distribution of myeloid and lymphoid lineage-biased subsets of HSCs in *Zeb1^−/−^* mice irrespective of altered *Zeb1^−/−^* HSC transcriptional programming, suggesting an important cell-intrinsic role for *Zeb1* specific to LMPP differentiation potential in vivo, operating independently of the influences of lineage-biased HSCs.

Other paradigms of HSC differentiation point to the functional heterogeneity of multi-potent progenitors that may influence the *Zeb1* regulation of lympho-myeloid differentiation. Researchers have identified four populations of multi-potent progenitors (MPP1-4) generated from HSCs, each with differing lineage bias potential [25,26]. MPP1 generates MPP2, which is myeloid/platelet-biased; MPP3, which is myeloid-biased, and MPP4, which is lymphoid-primed and overlaps considerably with LMPP function [27]. Of relevance to our study, we identified defects in both the lymphoid and monocyte/macrophage lineages after the transplantation of *Zeb1^−/−^* LMPPs that, as alluded to above, are progenitors more biased toward the lymphoid rather than myeloid lineage. Nonetheless, the observed reduction in granulocytic differentiation after *Zeb1^−/−^* HSC transplantation [11], but not seen following *Zeb1^−/−^* LMPP transplantation, suggests that *Zeb1*-dependent granulocytic differentiation in transplantation likely depends on other multi-potent progenitor subsets, including MPP2 and MPP3. Future studies should be directed at investigating the *Zeb1*-mediated regulation of MPP1-4 to specific hematopoietic lineages in transplantation, as well as to assess the contributions of MPP1-4 to *Zeb1*-mediated steady-state/native hematopoiesis using barcoding and lineage tracing technologies. It will be of considerable importance to delineate the role of *Zeb1* in the latter context, given that the prevailing dogma that HSCs contribute to steady-state/native hematopoiesis has been challenged, proffering instead that the major source of steady-stage blood production is the multi-potent progenitor pool [28,29].

Intriguingly, our data reveal defects in the engraftment of B-cell and macrophage lineages to the spleen, but not BM, in *Zeb1^−/−^* LMPPs after transplantation, with at least two possible explanations for this observation. First, while the overall homing/migratory capacity of *Zeb1^−/−^* LMPPs after transplantation appears to be unimpaired, as evidenced by equivalent engraftment during the first week (Figure 1D), it is possible that B-cell and macrophage lineages derived from *Zeb1^−/−^* LMPPs at later time points in transplantation develop *Zeb1* dependency for migration from the bone marrow to the spleen, congruent with the well-established role for *Zeb1* in cellular trafficking in other tissues and in the setting of cancer metastasis [8,30]. Second, the spleen is a site of maturation for both developing B cells and myeloid cells with inflammatory potential [31,32], and reduced PB engraftment in these lineages in *Zeb1^−/−^* LMPP transplant recipients may reflect a block in their maturation in the spleen. As we have shown the role of *Zeb1* in regulating the monocyte/macrophage lineage here and elsewhere [11], and the role of *Zeb1* in inflammation has been established [33], this hypothesis warrants further investigation in both steady-state hematopoiesis in lineage-specific conditional knockout models and transplantation. Parenthetically, impaired migration and lineage-specific maturation defects are both precepts that can be applied to explain the defective engraftment in *Zeb1^−/−^* LMPP-derived T cells during their development and maturation in the BM, spleen, and thymus.

The role of the closely related ZEB transcription factor, *Zeb2*, may also be pertinent when considering the impact of *Zeb1* mediated regulation of LMPP differentiation. Using conditional mouse models to knockout *Zeb2* during HSC development in utero (with *Tie2-Cre* and *Vav-Cre*) or in adult HSCs (using *Mx1-Cre*), *Zeb2* has been identified a critical regulator of hematopoietic cell differentiation [34,35]. In the adult hematopoietic system, mice engineered to be deficient in *Zeb2* in HSCs display an expansion of granulocytes, defects in erythroid, megakaryocytes, monocytes, and B-cells with unchanged T-cell abundance [35]. Except for the granulocyte lineage, *Zeb2^−/−^* HSCs also demonstrated a multi-lineage repopulation defect after transplantation [35]. Taken together with further studies exploring the genetic co-operation between *Zeb1* and *Zeb2* in hematopoiesis [13], these data suggest both distinct and overlapping functions for *Zeb1* and *Zeb2* during hematopoietic differentiation. Given that *Zeb1* functions mainly to sustain the overall integrity of HSCs [11] and that *Zeb2* appears to be more critical for multilineage differentiation than other HSC functions [13,35], future studies should investigate commonalities and differences between *Zeb1* and *Zeb2* mediated regulation of LMPP differentiation in vivo.

## Data Availability

Data will be made available on request.

## Supplementary Materials

The following supporting information can be downloaded at: https://www.mdpi.com/article/10.3390/biom13091386/s1, Figure S1: LMPP immunophenotyping gating strategy; Figure S2: Lineage biased HSC immunophenotyping.
