# Supplementary material for: Zeb1 Regulates the Function of Lympho-Myeloid Primed Progenitors after Transplantation
Source: Biomolecules. 2023 Sep 14;13(9):1386. doi: 10.3390/biom13091386 (PMC10526482; doi:10.3390/biom13091386)
Supplement: Supplementary file 1 [file biomolecules-13-01386-s001.zip › biomolecules-2506297-supplementary.pdf]

biomolecules

MDPI

Article

Zeb1 Regulates the Function of Lympho-Myeloid Primed Progenitors after Transplantation

Alhomidi Almotiri [1,2], Ashleigh S. Boyd [3,4] and Neil P. Rodrigues [2,*]

[1] Department of Clinical Laboratory Sciences, College of Applied Medical Sciences-Dawadmi, Shaqra University, Dawadmi 17464, Saudi Arabia; hsalmutiri@su.edu.sa
[2] European Cancer Stem Cell Research Institute, School of Biosciences, Cardiff University, Hadyn Ellis Building, Cardiff CF24 4HQ, UK
[3] Department of Surgical Biotechnology, Division of Surgery and Interventional Science, Royal Free Hospital, University College London, London NW3 2PS, UK; a.boyd@ucl.ac.uk
[4] Institute of Immunity and Transplantation, University College London, London NW3 2PP, UK
* Correspondence: rodriguesn@cardiff.ac.uk; Tel.: +44-29206-88507

Supplementary Figures

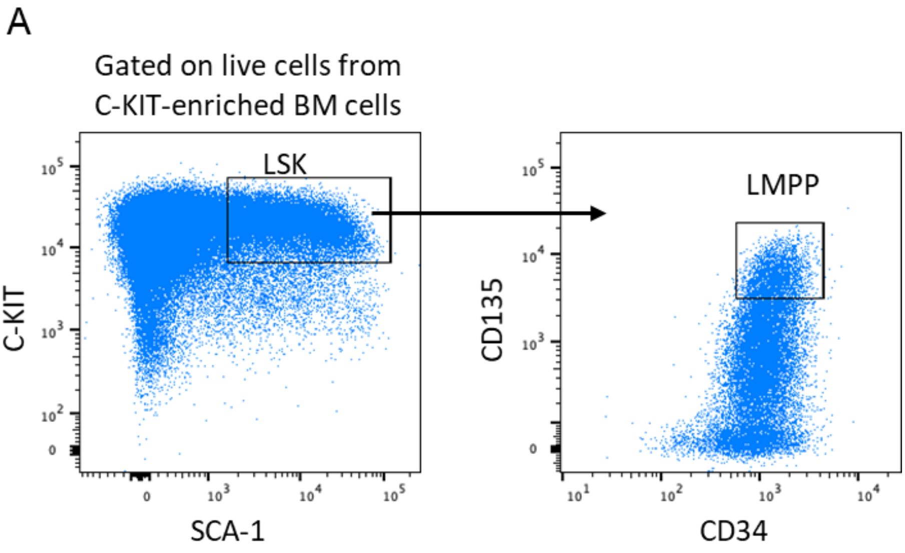

Figure S1. LMPP immunophenotyping gating strategy **(A)** Representative FACS plots showing the gating strategy of LMPP sorting for transplantation from C-KIT-enriched BM cells.

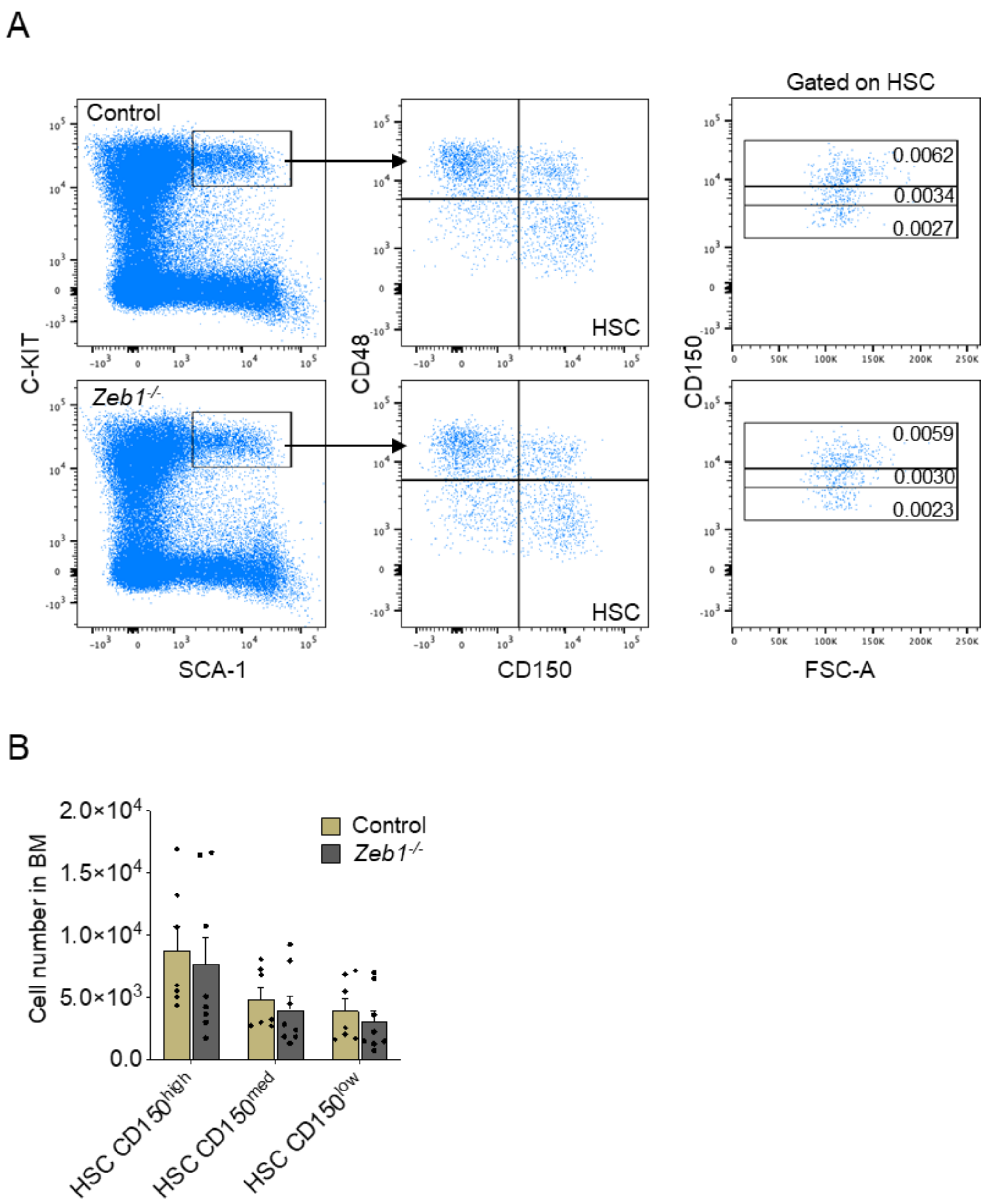

Figure S2. Lineage biased HSC immunophenotyping (A) Representative flow cytometry plots of the analysis of BM HSCs according to the expression of CD150 14 days after the last dose of pIpC. (B) Analysis of cell number of HSCs populations in the BM from control (n=7) and *Zeb1*$^{-/-}$ (n=8) mice from 3 independent experiments at D14 after the last pIpC dose.
